# META-ANALYSIS OF AARC AND EMOTIONAL AND PHYSICAL WELL-BEING

**DOI:** 10.1093/geroni/igz038.1412

**Published:** 2019-11-08

**Authors:** Serena Sabatini, Barbora Silarova, Anthony Martyr, Rachel Collins, Clive Ballard, Kaarin J Anstey, Sarang Kim, Linda Clare

**Affiliations:** 1 Centre for Research in Ageing and Cognitive Health, University of Exeter, Exeter, United Kingdom, University of Exeter, United Kingdom; 2 University of Exeter, Exeter, England, United Kingdom; 3 University of New South Wales, Sidney, New South Wales, Australia; 4 University of Tasmania, Hobart, Tasmania, Australia

## Abstract

Associations of awareness of age-related change (AARC) with emotional and physical well-being and cognitive functioning were synthesised in a systematic review with a correlational random-effects meta-analysis. Twelve studies were included in the review, nine exploring the association between AARC and emotional well-being and eleven exploring the association between AARC and physical well-being. No study explored the association between AARC and cognition. There is evidence of weak associations between higher level of AARC gains and better emotional well-being and between higher level of AARC losses and both poorer emotional well-being and poorer physical well-being. There was no association between AARC gains and physical well-being. There is some indication that AARC gains and losses can play a role in emotional well-being and that AARC losses are associated with physical well-being but these associations are weak. Due to the limited number of studies and their high heterogeneity, interpretation of these results remains unclear.

